# Severe acute respiratory syndrome coronavirus 2(SARS-CoV-2) infection during late pregnancy: a report of 18 patients from Wuhan, China

**DOI:** 10.1186/s12884-020-03026-3

**Published:** 2020-07-08

**Authors:** Lu Zhang, Lan Dong, Lei Ming, Min Wei, Jun Li, Ruheng Hu, Jing Yang

**Affiliations:** 1 Department Of Obstetrics and Gynecology, Renmin Hospital Of Wuhan University, Wuhan, 430060 China; 2 Department Of Reproductive Medicine Center, Hubei Clinic Research Center for Assisted Reproductive Technology and Embryonic Development, Renmin Hospital Of Wuhan University, Wuhan, 430060 China; 3 Department Of Obstetrics, The Central Hospital of Qianjiang City, Qianjiang, 433199 China

**Keywords:** Corona Virus Disease 2019(COVID-19), Severe Acute Respiratory Syndrome Coronavirus 2(SARS-COV-2), Neonatal pneumonia, Premature

## Abstract

**Background:**

Compared with Severe Acute Respiratory Syndrome (SARS) and Middle East Respiratory Syndrome (MERS), Corona Virus Disease 2019(COVID-19) spread more rapidly and widely. The population was generally susceptible. However, reports on pregnant women infected with SARS-CoV-2 were very limited. By sharing the clinical characteristics, treatments and outcomes of 18 patients with COVID-19 during late pregnancy, we hope to provide some references for obstetric treatment and management.

**Methods:**

A total of 18 patients with COVID-19 treated at Renmin Hospital of Wuhan University were collected. The epidemiological characteristics, clinical manifestations, laboratory tests, chest CT and pregnancy outcomes were performed for analysis.

**Results:**

1. 18 cases of late pregnancy infected with SARS-CoV-2 pneumonia were delivered at 35 ^+ 5^ weeks to 41 weeks. According to the clinical classification of COVID-19, 1 case was mild type, 16 cases were ordinary type, and 1 case was severe type. 2. According to imaging examinations: 15 (83%) cases showed unilateral or bilateral pneumonia, 2 (11%) cases had pulmonary infection with pleural effusion, and 1 (6%) case had no abnormal imaging changes. 8 (44%) cases were positive and 10 (56%) cases were negative for nasopharyngeal-swab tests of SARS-CoV-2. 3. Among the 18 newborns, there were 3 (17%) premature infants, 1 (6%) case of mild asphyxia, 5 (28%) cases of bacterial pneumonia, 1 (6%) case of gastrointestinal bleeding, 1 (6%) case of necrotizing enteritis, 2 (11%) cases of hyperbilirubinemia and 1 (6%) case of diarrhea. All the newborns were negative for the first throat swab test of SARS-CoV-2 after birth. 4. Follow-up to Mar 7, 2020, no maternal and neonatal deaths occurred.

**Conclusions:**

The majority of patients in late term pregnancy with COVID-19 were of ordinary type, and they were less likely to develop into critical pneumonia after early isolation and antiviral treatment. Vertical transmission of SARS-CoV-2 was not detected, but the proportion of neonatal bacterial pneumonia was higher than other neonatal diseases in newborns.

## Background

On Jan 30, 2020, the World Health Organization (WHO) listed the new coronavirus pneumonia epidemic as a public health emergency of international concern [1]. The WHO named the new coronavirus-infected disease as Corona Virus Disease 2019(COVID-19), and the International Virus Classification Commission named the new coronavirus as Severe Acute Respiratory Syndrome Coronavirus 2(SARS-CoV-2). The epidemic has spread across China and the world rapidly, posing a continuing threat to global health. Fever, cough, chest tightness, and dyspnea may appear in patients with COVID-19, however, acute respiratory distress syndrome and septic shock could lead to death [2, 3]. Great changes take place in the respiratory system during pregnancy due to changes in hormones and physiological structure [4, 5]. The immune system is tolerant to the paternal antigen from the fetus, and certain cell-mediated immunity is suppressed, which could make pregnant women more prone to critical illness after infected with the virus [6, 7]. At present, there are few reports about pregnant women with COVID-19 worldwide. Renmin Hospital of Wuhan University is one of the first batch of maternal health and critical illness for COVID-19 designated in Wuhan, China. From Jan 30, 2020, pregnant women with COVID-19 had been admitted to the hospital. The purpose of this article is to analyze the clinical characteristics and outcomes of 18 patients with late term pregnancy infected with COVID-19 retrospectively.

## Methods

### Patients

From Jan 30, 2020 to Mar 1, 2020, a total of 18 patients with COVID-19 in the 3rd trimester were clinically diagnosed (10 cases) or confirmed (8 cases) and hospitalized at Renmin Hospital of Wuhan University. Statistical analysis was performed on the age of women, gestational week of labor, source of infection, symptoms, blood tests, chest CT, throat swab tests of SARS-CoV-2, obstetric complications and neonatal outcomes. This study was approved by the institutional ethics board of Renmin Hospital of Wuhan University and the need for informed consent was waived (No. WDRY2020-K097).

### Diagnostic criteria

Reference to the “Notice on printing and distributing the diagnosis and treatment plan of pneumonia with new coronavirus infection (trial version 5)” issued by National Health Committee [8].

#### Suspected cases

Patients with any one history of epidemiology or no history of epidemiology, and in line with 2 of the clinical manifestations: (1) Epidemiological history 14 days prior to the onset of the disease, there was a history of travel or residence in the reported community in Wuhan and surrounding areas, or a history of contact with patients of COVID-19, or cluster disease. (2) Clinical manifestations Fever and/or respiratory symptoms, blood routines suggest that the total number of white blood cells is in the normal range or reduced, or the lymphocyte count is reduced in the early stage of the disease.

#### Clinically diagnosed cases

Those suspected cases with imaging features of novel coronavirus pneumonia.

#### Confirmed cases

Those with one of the following pathogenic evidence in suspected cases or clinically diagnosed cases:

1. real-time fluorescent RT-PCR of respiratory or blood samples detected positive for SARS-CoV-2 nucleic acid.
2. Viral gene sequencing of respiratory or blood samples shows highly homologous to known novel coronaviruses.

### Laboratory confirmation of SARS-CoV-2

A nucleic acid detection kit (Duplex fluorescence PCR methods, from Jiangsu Shuoshi Biotechnology) was used to detect SARS-CoV-2. This kit uses real-time fluorescent PCR technology and hydrolysis probe technology to allow the qualitative detection of one or more pathogens in the same reaction at the nucleic acid level.

### Clinical treatment

After admission, all pregnant women with COVID-19 in this study group were given with low-flow oxygen inhalation and finger oxygen saturation monitoring, oral oseltamivir/abidol and Lianhuaqingwen Capsules (Chinese traditional medicine), and ambroxol, IFN-α nebulized inhalation selectively. Patients with bacterial infections should use antibacterial drugs in combination with antiviral treatment as soon as possible, such as azithromycin, cephalosporin, moxifloxacin, etc. Breastfeeding should be suspended for 2 weeks after cesarean section or vaginal birth and antiviral therapy could be enhanced, such as ribavirin. Short term usage of glucocorticoids and gamma globulin could be considered based on the symptom of dyspnea and chest imaging progress.

### Statistical analysis

Using SPSS17.0 software, measurement data of normal distribution was expressed as mean ± standard deviation. All counts and measurement data were descriptive statistics, and the results were expressed as percentages.

## Results

### General information and clinical characteristics in patients of COVID-19 in the 3rd trimester (Table 1)

Eighteen patients with late term pregnancy, ages 24–34 years, delivered at 35^+5^weeks to 41 weeks. There were 2 (11%) cases infected followed by hospital visits, 3 (17%) cases by family members infected with COVID-19, 1 (6%) case by colleague infected with COVID-19, and 2 (11%) cases were occupational exposures due to medical staff. Another 10 (56%) cases without clear history of infection exposure had a clinical diagnosis of COVID-19 after admission. Most pregnant women had fever, cough, sore throat, fatigue, chest tightness or shortness of breath, diarrhea, and runny nose (Table 1). According to the clinical classification of the 5th edition of the guidelines [8], 1 (6%) patient was mild type, 16 (88%) patients were ordinary type, 1(6%) patient was severe type, but no critically ill patients. All the patients were cured and discharged.

**Table 1:** General information and clinical characteristics in 18 patients of COVID-19 in the 3rd trimester

Age (year)	
Mean (SD)	29.11 (2.78)
Range	24–34
Gestational age at illness (weeks)	
Mean (SD)	38.40 (1.56)
Range	(35.71–41)
Source of infection	
Hospital visit	2 (11%)
Household infected with SARS-CoV-2 pneumonia	3 (17%)
Colleague infected with SARS-CoV-2 pneumonia	1 (6%)
Occupational exposure	2 (11%)
Unknown	10 (55%)
Signs and symptoms of COVID-19 at admission	
Fever	5 (28%)
Cough	3 (17%)
Sore throat	1 (6%)
Fatigue	2 (11%)
Shortness of breath and chest tightness	2 (11%)
Diarrhea	1 (6%)
Runny nose and nasal congestion	1 (6%)
Clinical typing	
Mild	1 (6%)
Ordinary	16 (88%)
Severe	1 (6%)
Critical	0 (0%)

### Blood test results at admission in patients of COVID-19 in the 3rd trimester (Table 2)

Blood routing test: Leucocytes increased in 7 (39%) cases, neutrophils increased in 14 (78%) cases, lymphocyte count decreased in 8 (44%) cases, hemoglobin decreased in 4 (22%) cases. C-reactive protein (CRP) increased in 10 (56%) cases, and procalcitonin (PCT) was increased in 5 (28%) cases. Biochemical examination: Alanine aminotransferase (ALT) was above the normal range in 2 (11%) cases, aspartate aminotransferase (AST) was above the normal range in 3 (17%) cases, lactate dehydrogenase was above the normal range in 5 (28%) cases, serum creatinine, urea and creatine kinase were in the normal range.

**Table 2:** Blood test results at admission in 18 patients of COVID-19 in the 3rd trimester

Leucocytes (× 10^9^ /L; normal range 3·5–9·5)	9.51 (3.66)
Increased	7 (39%)
Neutrophils (%, normal range 40–75)	79.92 (6.86)
Increased	14 (78%)
Lymphocytes (× 10^9^ /L; normal range 1·1–3·2)	1.20 (0.29)
Decreased	8 (44%)
Hemoglobin (g/L; normal range 115–150)	124.89 (12.81)
Decreased	4 (22%)
Procalcitonin (ng/mL; normal range < 0.1)	0.13 (0.14)
Increased	5 (28%)
C-reactive protein (mg/L; normal range 0–10)*	19.01 (20.48)
Increased	10 (56%)
Alanine aminotransferase (U/L; normal range 7–40)	17.94 (13.17)
Increased	2 (11%)
Aspartate aminotransferase (U/L; normal range 13–35)	23.12 (9.92)
Increased	3 (17%)
Serum creatinine (μmol/L; normal range 41–73)	45.29 (7.74)
normal range	18 (100%)
Blood urea (mmol/L; normal range 2.6–7.5)	3.23 (0.68)
normal range	18 (100%)
Lactate dehydrogenase (U/L; normal range 120–250)	217.80 (38.65)
Increased	5 (28%)
Creatine kinase (U/L; normal range 40–200)	48.30 (31.75)
Normal range	18 (100%)

### Chest CT and the nucleic acid test of SARS-CoV-2 in patients of COVID-19 in the 3rd trimester (Table 3)

18 patients in this study had completed chest CT before admission. The results of CT revealed 9 (50%) cases of unilateral pneumonia, 6 (33%) cases of bilateral pneumonia, 2 (11%) cases of pulmonary infection adjoint of pleural effusion. No abnormal imaging changes were found in 1 (6%) case (the nucleic acid test of SARS-CoV-2 was positive). The chest CT of 17 pregnant women were mostly in line with the imaging characteristics of typical COVID-19 patients: multiple lamellar, patchy, segmental ground glass shadows in the single or double lungs, mainly distributed outside the band. 8 (44%) patients were positive and 10 (56%) patients were negative for nasopharyngeal-swab tests of SARS-CoV-2.

**Table 3:** Chest CT and new coronavirus nucleic acid tests in 18 patients of COVID-19 in the 3rd trimester

Chest CT	
Unilateral pneumonia	9 (50%)
Bilateral pneumonia	6 (33%)
Pneumonia with pleural effusion	2 (11%)
No abnormal imaging changes	1 (6%)
Nucleic acid test of SARS-CoV-2 for pregnancy women	
Positive	8 (44%)
Negative	10 (56%)

### Pregnancy outcomes in patients of COVID-19 in the 3rd trimester (Table 4)

Of the 18 pregnant women, 17 (94%) patients were delivered by cesarean section, and 1 (6%) patient was vaginal delivery. There were 4 (22%) cases of premature rupture of membranes, 2 (11%) cases of scarred uterus, 1 (6%) case of severe preeclampsia, 3 (17%) cases of gestational diabetes, 1 (6%) case of fetal distress, and 2 (11%) cases of meconium-stained amniotic fluid. There were 2 (11%) cases of B-Lynch or other compression sutures due to uterine contraction fatigue during operations, and 1 (6%) case of postpartum hemorrhage. In 18 newborns, the first nucleic acid test of SARS-CoV-2 were negative within 24 h and the weight range was 2300-3910 g. There were 3 (17%) cases of premature infants, 1 (6%) case of mild neonatal asphyxia, 1 (6%) case of neonatal gastrointestinal bleeding, 1 (6%) case of necrotizing enteritis, 2 (11%) cases of hyperbilirubinemia, and 1 (6%) case of newborn diarrhea. 5 (28%) cases were diagnosed with bacterial pneumonia, based on the history of symptoms, blood test, procalcitonin, C-reactive protein, sputum culture and radiology examination and all returned to normal after anti-inflammatory treatment. 18 infants were discharged after quarantine. Followed up to Mar 7, 2020, no neonatal death occurred.

**Table 4:** Pregnancy outcomes in 18 patients of COVID-19 in the 3rd trimester

Pregnancy complications	
Premature rupture of membranes	4 (22%)
Scar uterus	2 (11%)
Severe preeclampsia	1 (6%)
Gestational diabetes	3 (17%)
Fetal distress	1 (6%)
Meconium-stained amniotic fluid	2 (11%)
B-Lynch or other compression sutures	2 (11%)
Postpartum hemorrhage	1 (6%)
Delivery method	
Vaginal delivery	1 (6%)
Cesarean section	17 (94%)
Neonatal outcomes	
Premature birth	3 (17%)
Full term birth	15 (83%)
Birth weight	
Mean (SD)	3181.11 (448.63)
Range (gram)	2300–3910
The first nucleic acid test of SARS-CoV-2 for neonates	
Negative	18 (100%)
Positive	0 (0%)
Mild neonatal asphyxia	1 (6%)
Chest radiograph and blood test results suggest bacterial pneumonia	5 (28%)
Neonatal gastrointestinal bleeding	1 (6%)
Neonatal necrotizing enteritis	1 (6%)
Neonatal hyperbilirubinemia	2 (11%)
Neonatal diarrhea	1 (6%)

## Discussion

Among the pregnant women admitted to our hospital in late pregnancy, 1 case was mild type, 16 cases were ordinary type, and 1 case was severe type. The clinical manifestations included fever, cough, sputum, fatigue, chest tightness, shortness of breath, and diarrhea. According to epidemiological statistics, 6 cases had a history of contact with patients of COVID-19, 2 cases of hospital visits, and the remaining 10 patients was not clear about the source of the infection. The peripheral blood leukocytes levels of these pregnant women could be normal, but for a subset of patients they were elevated during bacterial infection. Decrease in lymphocyte counts was also observed in certain patients. ALT, AST, and LDH were increased in some patients. However, serum creatinine, urea, and creatine kinase were within normal range in all the patients. Chest CT of patients with COVID-19 had certain characteristics, and early manifestations were multiple flaky ground-glass shadows [9]. Most patients in this study had typical CT manifestations similar to the general population: multiple flaky, patchy, segmental ground glass shadows in one or both lungs, mainly distributed outside the band. Two patients had pleural effusion in addition to typical CT findings. One patient had occasional cough and denied a history of exposure to COVID-19. Her blood routine showed that white blood cells were in normal range, but lymphocyte count decreased. There was no abnormal radiographic change in CT examination, but the nucleic acid test of SARS-CoV-2 was positive. Therefore, in areas with a high incidence of epidemic cases, patients highly suspected of COVID-19 with no characteristic changes in CT imaging should require further testing with throat swabs. A normal CT result cannot be used as an exclusion criterion for COVID-19.

In this study, 18 patients terminated pregnancy during the 3rd trimester, and the gestational age was from 35^+ 5^ to 41 weeks. 13 cases were terminated by cesarean section because of labor onset, premature rupture of membranes, severe preeclampsia, scar uterus, and fetal distress. 3 cases of pneumonia did not improve significantly after antiviral therapy, and thus cesarean section performed. 1 case requested for caesarean section at 39^+ 5^ weeks due to social and psychosocial factors. In addition, 1 case was vaginal delivery. Under strict isolation and active treatment, all the patients had been cured and discharged. Before the outbreak of SARS-CoV-2 pneumonia, the cesarean section rate in the hospital was 48–52%. The indications for cesarean section were scarred uterus, placenta previa, dystocia, multiple pregnancy, preeclampsia, fetal distress, etc. We considered that the indications for cesarean section of pregnancy with COVID-19 should still be based on obstetric management principles. For the patients of mild or ordinary pneumonia, the gestational age could be prolonged properly under monitoring of changes in pneumonia and fetal. If the mother’s condition did not improve and the gestational age of the newborn was assessed to have a certain survival rate, early delivery was beneficial to improve COVID-19 maternal lung ventilation. For patients who are expected to deliver in a short time, we should make preparations for delivery immediately. When closely monitoring mother, fetus and the progress of labor, midwifery personnel should make III-level protective measures to avoid infection to the greatest extent.

SARS-CoV-2 was a novel coronavirus that is pathogenic to humans. Roujian Lu et al. found that SARS-CoV-2 had a similar structure of receptor binding domain as SARS-CoV-1, which indicated that SARS-CoV-2 infection might have a similar pathogenesis as SARS-CoV-1 infection [10, 11]. Some scholars speculated that the risk of vertical transmission of SARS-CoV-2 might be as low as SARS-CoV-1 [12]. The clinical data of 9 pregnant women were analyzed by Zhongnan Hospital of Wuhan University. The nucleic acid tests for SARS-CoV-2 were negative in the amniotic fluid, umbilical cord blood, neonatal pharynx, and breast milk of 6 patients. They believed that the vertical transmission of the SARS-CoV-2 was relatively low [13]. Also, some researchers performed pathological analysis of placenta in 3 pregnant women infected with SARS-CoV-2 in late pregnancy. Histopathological analysis of the placenta revealed 1 case with chorionic hemangioma and another with massive placental infarction. In all 3 cases, various degrees of fibrin deposition inside and around the villi with local syncytia nodules increased. Both placental tissue and neonatal throat swabs tested for SARS-CoV-2 were negative. They concluded that there was no pathological evidence of intrauterine vertical transmission of SARS-CoV-2 [14]. A study of various types of cells at the maternal-fetal interface found that the angiotensin-converting enzyme 2(ACE2), which was the receptor of SARS-CoV-2, expressed at low levels. Furthermore, it suggested that the maternal-fetal interface might not have a potential subgroup of susceptible cells of SARS-CoV-2, and SARS-CoV-2 infection during pregnancy did not cause intrauterine infection through vertical transmission of the placenta [15]. The results of our study was consistent with previous reports, and no vertical transmission of the SARS-CoV-2 was found. However, our study had certain limitations. We only performed swab nucleic acid testing in 18 neonates. No nucleic acid tests were performed on breast milk, placenta, amniotic fluid, and cord blood of pregnant women. Therefore, it is very important to collect evidence for the vertical transmission in the later stage. Additionally, it would be more valuable to evaluate the risk of the vertical transmission based on the data of multiple hospitals.

The outcomes of 18 neonates were as follows: 3 cases of premature infants, 1 case of mild neonatal asphyxia, 5 cases of bacterial pneumonia, 1 case of neonatal gastrointestinal bleeding, 1 case of necrotizing enteritis, 2 cases of hyperbilirubinemia, 1 case of neonatal diarrhea. Among the mothers of 5 newborns with bacterial pneumonia, 4 mothers tested positive for nucleic acid of SARS-CoV-2. The chest radiographs of these 5 neonates showed pneumonia lesion absorption after antibiotic treatment, and they had been cured. The occurrence of perinatal neonatal pneumonia was related to premature rupture of membranes, maternal infection and premature birth [16]. Our study found that the incidence of neonatal bacterial pneumonia was significantly higher than other neonatal diseases when the mother infected with COVID-19 was in the state of inflammatory stress or fever.

Twelve pregnant women were infected with SARS in 2003, and 3 of them died of respiratory failure or infection. Spontaneous abortion occurred in 4 of the 7 early pregnancy patients. In the second or third trimester of pregnancy, 3 of the 5 patients gave birth at 26–32 weeks, and 2 gave birth at 33 weeks and term [17, 18]. Sarah H [19] summarized the cases of pregnant women with MERS-CoV in the MEDLINE database from 2012 to 2016, of which 2 cases were reported by her. Of all 11 patients, 6 patients were admitted to the ICU, 3 patients died during hospitalization, and the infant mortality rate was 27%. The mortality rate of 11 pregnant women infected with MERS-CoV was not statistically different from the 35% total mortality rate. Compared with previous reports of SARS and MERS, our research included a higher number of pregnancy women with COVID-19, but the maternal and infant outcomes were relatively better. The clinical classification of patients were mostly ordinary type, and the possibility of progress to severe or critical novel coronavirus pneumonia had been reduced after early isolation and drug treatments. However, we should still be vigilant and pay more attention to the monitoring and treatment of pregnant women infected with SARS-CoV-2 pneumonia, and control the disease towards severe infection, ARDS and multiple organ failure which endanger the lives of both the mothers and fetus.

## Conclusions

We summarized the recent managements and treatments of these late pregnancy patients of COVID-19 and believe that the mild or ordinary patients who have not reached full-term could continue pregnancy if the treatments are effective. However, when the mother’s condition of pneumonia is aggravated, cesarean section should be preferred to end the delivery to relieve the cardiopulmonary burden in the labor process, and a more effective therapy for viral pneumonia should be given after the surgery to give priority to ensuring the mother’s life safety. The small number of patients in this study has certain limitations. By observing the pregnancy process of patients with COVID-19 in the early, middle and late stages and long-term follow-up of infants, we will fully understand the risks of pregnancy associated with COVID-19.

## Data Availability

The data that support the findings of this study are available from the corresponding author but restrictions apply to the availability of these data, which were used under license for the current study, and so are not publicly available. Data and materials in the current study are available from the corresponding author upon reasonable request and permission.

## Abbreviations

SARS-CoV-2: Severe Acute Respiratory Syndrome Coronavirus 2
SARS: Severe Acute Respiratory Syndrome
WHO: World Health Organization
MERS: Middle East Respiratory Syndrome
COVID-19: Corona Virus Disease 2019
ACE2: Angiotensin-Converting Enzyme 2
